# 2,17-Dichloro-8,9,10,11-tetra­hydro-19*H*-dibenzo[*k*,*n*][1,10,4,7]dioxadiaza­cyclo­penta­decine-7,12(6*H*,13*H*)-dione

**DOI:** 10.1107/S1600536812020351

**Published:** 2012-05-12

**Authors:** Michaela Pojarová, Michal Dušek, Zdeňka Sedláková, Emanuel Makrlík

**Affiliations:** a Institute of Physics, AS CR, v.v.i., Na Slovance 2, 182 21 Prague 8, Czech Republic; b Institute of Macromolecular Chemistry, AS CR v.v.i., Heyrovského nám. 2, 162 06 Prague 6, Czech Republic; c Faculty of Environmental Sciences, Czech University of Life Sciences, Prague, Kamýcká 129, 165 21 Prague 6, Czech Republic

## Abstract

In the crystal structure of the title compound, C_19_H_18_Cl_2_N_2_O_4_, N—H⋯O hydrogen bonds link the mol­ecules into infinite chains along the *b* axis. The structure also features weak C—H⋯O and C—H⋯Cl hydrogen bonds and C—H⋯π and (lone pair)⋯π inter­actions [Cl⋯centroid = 3.5871 (7) Å]. An intra­molecular N—H⋯O bond occurs.

## Related literature

For the synthesis, see: Ertul *et al.* (2009 ▶). For applications of macrocycles, see: Hayvali & Hayvali (2005 ▶); Kleinpeter *et al.* (1997 ▶); Jaiyu *et al.* (2007 ▶); Christensen *et al.* (1997 ▶); Alexander (1995 ▶).
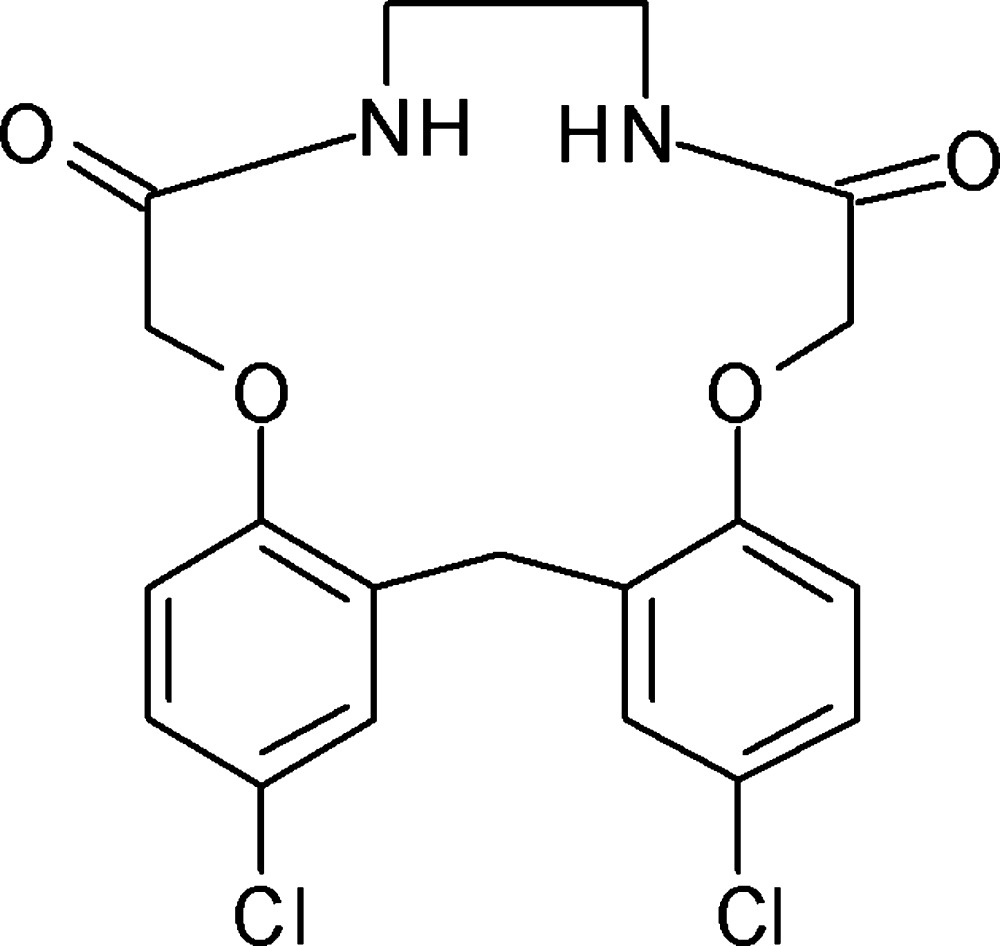


## Experimental

### 

#### Crystal data

C_19_H_18_Cl_2_N_2_O_4_
*M* *_r_* = 409.25
Monoclinic, 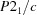

*a* = 12.0877 (3) Å
*b* = 8.73462 (15) Å
*c* = 17.3712 (4) Å
β = 93.588 (2)°
*V* = 1830.48 (7) Å^3^
*Z* = 4
Cu *K*α radiation
μ = 3.44 mm^−1^
*T* = 120 K
0.31 × 0.22 × 0.21 mm

#### Data collection

Agilent Xcalibur Atlas Gemini ultra diffractometer
Absorption correction: analytical (*CrysAlis PRO*; Agilent, 2010 ▶) *T*_min_ = 0.175, *T*_max_ = 0.342
19596 measured reflections
3271 independent reflections
3210 reflections with *I* > 2σ(*I*)
*R*_int_ = 0.028

#### Refinement

*R*[*F* ^2^ > 2σ(*F* ^2^)] = 0.029
*wR*(*F* ^2^) = 0.074
*S* = 1.07
3271 reflections
244 parameters
2 restraints
H-atom parameters constrained
Δρ_max_ = 0.20 e Å^−3^
Δρ_min_ = −0.29 e Å^−3^

### 

Data collection: *CrysAlis PRO* (Agilent, 2010 ▶); cell refinement: *CrysAlis PRO*; data reduction: *CrysAlis PRO*; program(s) used to solve structure: *SHELXS97* (Sheldrick, 2008 ▶); program(s) used to refine structure: *SHELXL97* (Sheldrick, 2008 ▶); molecular graphics: Mercury (Macrae *et al.*, 2006 ▶) and *ORTEP-3* (Farrugia, 1997 ▶); software used to prepare material for publication: *publCIF* (Westrip, 2010 ▶).

## Supplementary Material

Crystal structure: contains datablock(s) I, global. DOI: 10.1107/S1600536812020351/aa2058sup1.cif

Structure factors: contains datablock(s) I. DOI: 10.1107/S1600536812020351/aa2058Isup2.hkl

Additional supplementary materials:  crystallographic information; 3D view; checkCIF report

## Figures and Tables

**Table 1:** Hydrogen-bond geometry (Å, °) *Cg*1 is the centroid of the C1–C6 ring.

*D*—H⋯*A*	*D*—H	H⋯*A*	*D*⋯*A*	*D*—H⋯*A*
N2—H1*N*2⋯O2^i^	0.89	2.05	2.8945 (15)	158
C14—H14*A*⋯O3^i^	0.97	2.54	3.5026 (17)	172
C14—H14*B*⋯O3^ii^	0.97	2.34	3.2934 (17)	167
C16—H16*B*⋯O1^iii^	0.97	2.59	3.2843 (17)	129
C19—H19*A*⋯Cl2^iv^	0.97	2.81	3.6201 (14)	142
C9—H9⋯*Cg*1^v^	0.93	2.88	3.8006 (14)	174
N1—H1*N*1⋯O1	0.90	2.16	2.5935 (15)	109

Symmetry codes: (i) 
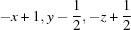
; (ii) 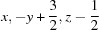; (iii) 
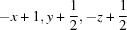
; (iv) 
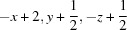
; (v) 
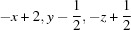
.

**Fig. 1.**
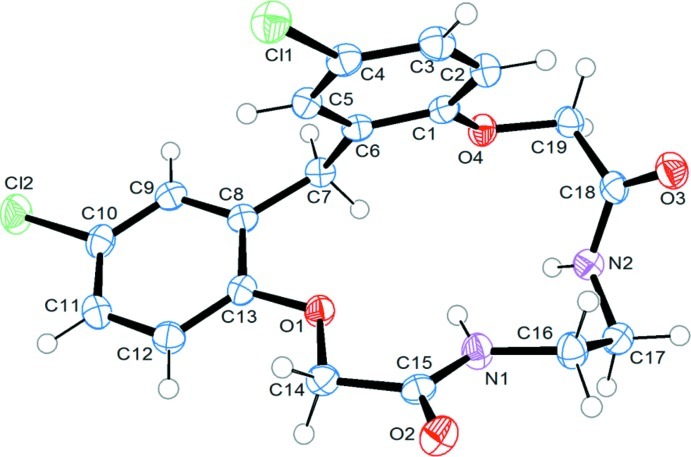
View of the title compound, together with atom-labelling scheme. Displacement ellipsoids are shown at the 50% probability level.

**Fig. 2.**
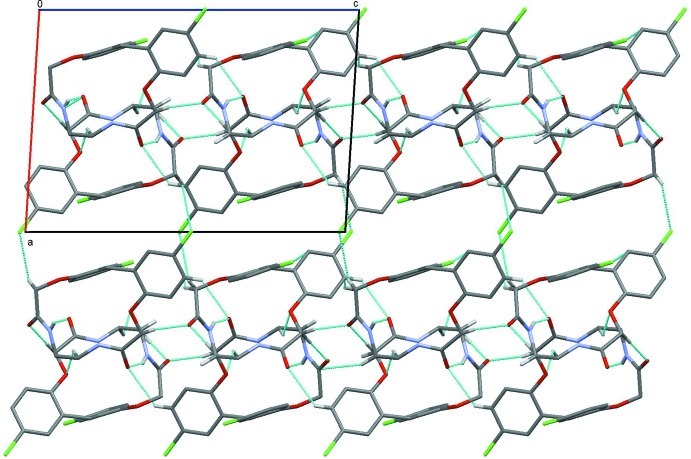
Projection along the b axis with highlighted hydrogen bonds between the molecules.

## supplementary crystallographic information

### Comment

Polyazalactones together with polyoxalactones and polyethers are studied for their ability to act as multidentate ligands and to complex various cations. Polyazalactones can incorporate transition metals into their cavities *via* an ion-dipole interaction (Hayvali *et al.*, 2005; Kleinpeter *et al.*, 1997). They are studied for their role in bioprocesses, catalysis, material science, and transport and separation (Jaiyu *et al.*, 2007; Christensen *et al.*, 1997; Alexander, 1995). In this paper, we report a crystal structure of lactam ionophore (Fig. 1 and Scheme). The macrocycle consists of two phenyl rings substituted with chlorine atom in *para* position. The neighbouring molecules are connected *via* hydrogen bonds between amide groups (Fig. 1 and Table 1). Weaker hydrogen bonds can be found between methylene groups and oxygen or chlorine atoms. The arrangement of the molecules in the crystal is influenced by the C—H···π interactions between the aromatic rings (C9—H9··· C1→C6 (*Cg*1)) and lone pair···π interaction between the chlorine atom Cl1 and neighbouring aromatic ring C8→C13(*Cg*2) (the distance between Cl1 and *Cg*2 is 3.5871 (7) Å).

### Experimental

All chemicals used were purchased from Fluka and used without further purification. The title compound was synthesized by means of method published by Ertul *et al.* (2009). Crystals were prepared by slow evaporation from methanol.

### Refinement

H atoms bound to C atoms were positioned geometrically and refined as riding with C—H distances 0.93–0.97 Å. H atoms bound to N atoms were located in a difference map and refined as riding with N—H bond restrained to 0.89 Å. The isotropic temperature parameters of all hydrogen atoms were calculated as 1.2**U*_eq_ of the parent atom.

### Crystal data

**Table:**

C_19_H_18_Cl_2_N_2_O_4_	*F*(000) = 848
*M**_r_* = 409.25	*D*_x_ = 1.484 Mg m^−^^3^
Monoclinic, *P*2_1_/*c*	Cu *K*α radiation, λ = 1.5418 Å
Hall symbol: -P 2ybc	Cell parameters from 16650 reflections
*a* = 12.0877 (3) Å	θ = 3.7–67.1°
*b* = 8.73462 (15) Å	µ = 3.44 mm^−^^1^
*c* = 17.3712 (4) Å	*T* = 120 K
β = 93.588 (2)°	Prism, colourless
*V* = 1830.48 (7) Å^3^	0.31 × 0.22 × 0.21 mm
*Z* = 4	

### Data collection

**Table:**

Agilent Xcalibur Atlas Gemini ultra diffractometer	3271 independent reflections
Radiation source: Enhance Ultra (Cu) X-ray Source	3210 reflections with *I* > 2σ(*I*)
Mirror monochromator	*R*_int_ = 0.028
Detector resolution: 10.3784 pixels mm^-1^	θ_max_ = 67.1°, θ_min_ = 3.7°
Rotation method data acquisition using ω scans	*h* = −14→14
Absorption correction: analytical (*CrysAlis PRO*; Agilent, 2010)	*k* = −8→10
*T*_min_ = 0.175, *T*_max_ = 0.342	*l* = −20→20
19596 measured reflections	

### Refinement

**Table:**

Refinement on *F*^2^	Primary atom site location: structure-invariant direct methods
Least-squares matrix: full	Secondary atom site location: difference Fourier map
*R*[*F*^2^ > 2σ(*F*^2^)] = 0.029	Hydrogen site location: inferred from neighbouring sites
*wR*(*F*^2^) = 0.074	H-atom parameters constrained
*S* = 1.07	*w* = 1/[σ^2^(*F*_o_^2^) + (0.0376*P*)^2^ + 0.7612*P*] where *P* = (*F*_o_^2^ + 2*F*_c_^2^)/3
3271 reflections	(Δ/σ)_max_ = 0.001
244 parameters	Δρ_max_ = 0.20 e Å^−^^3^
2 restraints	Δρ_min_ = −0.29 e Å^−^^3^

### Special details

**Table:**

Geometry. All s.u.'s (except the s.u. in the dihedral angle between two l.s. planes) are estimated using the full covariance matrix. The cell s.u.'s are taken into account individually in the estimation of s.u.'s in distances, angles and torsion angles; correlations between s.u.'s in cell parameters are only used when they are defined by crystal symmetry. An approximate (isotropic) treatment of cell s.u.'s is used for estimating s.u.'s involving l.s. planes.
Refinement. Refinement of *F*^2^ against ALL reflections. The weighted *R*-factor *wR* and goodness of fit *S* are based on *F*^2^, conventional *R*-factors *R* are based on *F*, with *F* set to zero for negative *F*^2^. The threshold expression of *F*^2^ > σ(*F*^2^) is used only for calculating *R*-factors(gt) *etc*. and is not relevant to the choice of reflections for refinement. *R*-factors based on *F*^2^ are statistically about twice as large as those based on *F*, and *R*- factors based on ALL data will be even larger. The H atoms were all located in a difference map, but those attached to carbon atoms were repositioned geometrically. The distance between hydrogen atoms and nitrogen atoms was restrained. The bond length was set to 0.87 Å with σ 0.02. The isotropic temperature parameters of hydrogen atoms were calculated as 1.2**U*_eq_ of the parent atom.

### Fractional atomic coordinates and isotropic or equivalent isotropic displacement parameters (Å^2^)

**Table:**

	*x*	*y*	*z*	*U*_iso_*/*U*_eq_	
Cl1	0.86335 (3)	1.01411 (4)	0.16078 (2)	0.03147 (11)	
Cl2	1.00225 (3)	0.24819 (5)	−0.01843 (2)	0.03989 (12)	
O4	0.78538 (8)	0.52610 (11)	0.38371 (5)	0.0251 (2)	
O1	0.64125 (7)	0.49162 (11)	0.15585 (5)	0.0248 (2)	
O3	0.58083 (8)	0.72265 (11)	0.47012 (6)	0.0306 (2)	
O2	0.39104 (8)	0.69986 (11)	0.15386 (6)	0.0291 (2)	
N2	0.55581 (9)	0.49941 (13)	0.40482 (6)	0.0247 (2)	
H1N2	0.5904	0.4132	0.3923	0.030*	
C19	0.74034 (11)	0.56132 (16)	0.45584 (8)	0.0262 (3)	
H19A	0.7809	0.6463	0.4799	0.031*	
H19B	0.7490	0.4736	0.4899	0.031*	
C3	0.79942 (11)	0.91308 (16)	0.29832 (8)	0.0269 (3)	
H3	0.7880	1.0154	0.3102	0.032*	
C5	0.84978 (11)	0.71987 (16)	0.20783 (8)	0.0241 (3)	
H5	0.8732	0.6951	0.1594	0.029*	
C11	0.79350 (12)	0.34870 (16)	−0.00686 (8)	0.0276 (3)	
H11	0.7834	0.3274	−0.0593	0.033*	
C8	0.82559 (11)	0.40890 (14)	0.15178 (8)	0.0222 (3)	
C1	0.79727 (10)	0.64599 (15)	0.33323 (8)	0.0230 (3)	
C6	0.82934 (10)	0.60427 (15)	0.25987 (8)	0.0223 (3)	
C7	0.83966 (11)	0.43695 (15)	0.23779 (8)	0.0237 (3)	
H7A	0.9119	0.3998	0.2567	0.028*	
H7B	0.7842	0.3784	0.2631	0.028*	
C13	0.72294 (11)	0.43609 (15)	0.11165 (8)	0.0228 (3)	
C9	0.91159 (11)	0.35413 (15)	0.11042 (8)	0.0248 (3)	
H9	0.9809	0.3374	0.1352	0.030*	
C12	0.70686 (11)	0.40584 (16)	0.03341 (8)	0.0268 (3)	
H12	0.6381	0.4238	0.0079	0.032*	
N1	0.49166 (10)	0.57462 (14)	0.24811 (7)	0.0271 (3)	
H1N1	0.5560	0.5268	0.2600	0.033*	
C15	0.46922 (10)	0.61525 (15)	0.17506 (8)	0.0230 (3)	
C14	0.54225 (11)	0.54793 (16)	0.11649 (8)	0.0242 (3)	
H14A	0.5038	0.4652	0.0889	0.029*	
H14B	0.5604	0.6256	0.0794	0.029*	
C18	0.61793 (11)	0.60295 (15)	0.44482 (7)	0.0242 (3)	
C2	0.78030 (11)	0.79873 (16)	0.35175 (8)	0.0263 (3)	
H2	0.7562	0.8245	0.3999	0.032*	
C4	0.83550 (11)	0.87211 (16)	0.22751 (8)	0.0250 (3)	
C17	0.44063 (11)	0.52947 (16)	0.38020 (8)	0.0270 (3)	
H17A	0.4034	0.5749	0.4225	0.032*	
H17B	0.4038	0.4337	0.3666	0.032*	
C10	0.89470 (11)	0.32410 (16)	0.03211 (8)	0.0268 (3)	
C16	0.43191 (11)	0.63655 (16)	0.31133 (8)	0.0276 (3)	
H16A	0.3546	0.6506	0.2945	0.033*	
H16B	0.4624	0.7357	0.3262	0.033*	

### Atomic displacement parameters (Å^2^)

**Table:**

	*U*^11^	*U*^22^	*U*^33^	*U*^12^	*U*^13^	*U*^23^
Cl1	0.0375 (2)	0.02398 (18)	0.0333 (2)	−0.00168 (13)	0.00512 (14)	0.00670 (13)
Cl2	0.0333 (2)	0.0531 (2)	0.0343 (2)	0.01109 (16)	0.00956 (15)	−0.00592 (16)
O4	0.0283 (5)	0.0241 (5)	0.0232 (5)	0.0001 (4)	0.0043 (4)	0.0026 (4)
O1	0.0209 (5)	0.0307 (5)	0.0227 (5)	0.0044 (4)	0.0010 (4)	0.0008 (4)
O3	0.0355 (5)	0.0277 (5)	0.0291 (5)	0.0002 (4)	0.0056 (4)	−0.0042 (4)
O2	0.0259 (5)	0.0293 (5)	0.0323 (5)	0.0061 (4)	0.0027 (4)	0.0076 (4)
N2	0.0253 (6)	0.0234 (6)	0.0255 (6)	0.0011 (4)	0.0013 (5)	0.0001 (4)
C19	0.0294 (7)	0.0287 (7)	0.0205 (6)	−0.0023 (6)	0.0020 (5)	0.0012 (5)
C3	0.0283 (7)	0.0210 (7)	0.0313 (7)	0.0004 (5)	0.0005 (5)	−0.0014 (5)
C5	0.0233 (6)	0.0260 (7)	0.0232 (6)	−0.0011 (5)	0.0025 (5)	0.0002 (5)
C11	0.0333 (7)	0.0268 (7)	0.0227 (7)	0.0004 (6)	0.0034 (5)	0.0003 (5)
C8	0.0246 (6)	0.0161 (6)	0.0258 (7)	−0.0012 (5)	0.0014 (5)	0.0019 (5)
C1	0.0198 (6)	0.0244 (7)	0.0245 (6)	−0.0019 (5)	−0.0006 (5)	0.0030 (5)
C6	0.0181 (6)	0.0226 (6)	0.0259 (7)	−0.0004 (5)	−0.0008 (5)	0.0004 (5)
C7	0.0239 (6)	0.0224 (6)	0.0248 (7)	0.0012 (5)	0.0004 (5)	0.0013 (5)
C13	0.0235 (6)	0.0201 (6)	0.0251 (7)	0.0004 (5)	0.0041 (5)	0.0011 (5)
C9	0.0231 (6)	0.0213 (6)	0.0299 (7)	−0.0002 (5)	0.0015 (5)	0.0019 (5)
C12	0.0257 (7)	0.0284 (7)	0.0261 (7)	0.0022 (5)	−0.0001 (5)	0.0016 (5)
N1	0.0258 (6)	0.0309 (6)	0.0247 (6)	0.0087 (5)	0.0016 (4)	0.0007 (5)
C15	0.0211 (6)	0.0195 (6)	0.0283 (7)	−0.0029 (5)	0.0007 (5)	0.0018 (5)
C14	0.0222 (6)	0.0256 (7)	0.0244 (7)	0.0016 (5)	−0.0008 (5)	0.0036 (5)
C18	0.0301 (7)	0.0246 (7)	0.0183 (6)	−0.0015 (5)	0.0042 (5)	0.0030 (5)
C2	0.0267 (7)	0.0278 (7)	0.0248 (7)	0.0005 (5)	0.0034 (5)	−0.0028 (6)
C4	0.0240 (6)	0.0231 (7)	0.0278 (7)	−0.0014 (5)	0.0001 (5)	0.0045 (5)
C17	0.0244 (7)	0.0300 (7)	0.0268 (7)	−0.0005 (5)	0.0031 (5)	0.0003 (6)
C10	0.0279 (7)	0.0237 (7)	0.0295 (7)	0.0020 (5)	0.0082 (5)	0.0001 (5)
C16	0.0277 (7)	0.0292 (7)	0.0262 (7)	0.0057 (6)	0.0030 (5)	−0.0006 (6)

### Geometric parameters (Å, º)

**Table:**

Cl1—C4	1.7451 (14)	C8—C13	1.4050 (18)
Cl2—C10	1.7448 (14)	C8—C7	1.5132 (18)
O4—C1	1.3794 (16)	C1—C2	1.391 (2)
O4—C19	1.4306 (16)	C1—C6	1.4031 (19)
O1—C13	1.3766 (16)	C6—C7	1.5185 (19)
O1—C14	1.4282 (15)	C7—H7A	0.9700
O3—C18	1.2300 (17)	C7—H7B	0.9700
O2—C15	1.2377 (16)	C13—C12	1.3864 (19)
N2—C18	1.3417 (18)	C9—C10	1.388 (2)
N2—C17	1.4548 (17)	C9—H9	0.9300
N2—H1N2	0.8948	C12—H12	0.9300
C19—C18	1.5243 (19)	N1—C15	1.3292 (18)
C19—H19A	0.9700	N1—C16	1.4560 (18)
C19—H19B	0.9700	N1—H1N1	0.8962
C3—C4	1.378 (2)	C15—C14	1.5077 (19)
C3—C2	1.393 (2)	C14—H14A	0.9700
C3—H3	0.9300	C14—H14B	0.9700
C5—C6	1.3880 (19)	C2—H2	0.9300
C5—C4	1.387 (2)	C17—C16	1.5171 (19)
C5—H5	0.9300	C17—H17A	0.9700
C11—C10	1.377 (2)	C17—H17B	0.9700
C11—C12	1.388 (2)	C16—H16A	0.9700
C11—H11	0.9300	C16—H16B	0.9700
C8—C9	1.3856 (19)		
			
C1—O4—C19	117.00 (10)	C10—C9—H9	119.9
C13—O1—C14	117.60 (10)	C13—C12—C11	119.91 (13)
C18—N2—C17	121.67 (12)	C13—C12—H12	120.0
C18—N2—H1N2	116.0	C11—C12—H12	120.0
C17—N2—H1N2	122.3	C15—N1—C16	122.65 (11)
O4—C19—C18	111.17 (10)	C15—N1—H1N1	117.8
O4—C19—H19A	109.4	C16—N1—H1N1	117.7
C18—C19—H19A	109.4	O2—C15—N1	123.36 (12)
O4—C19—H19B	109.4	O2—C15—C14	120.06 (12)
C18—C19—H19B	109.4	N1—C15—C14	116.55 (11)
H19A—C19—H19B	108.0	O1—C14—C15	108.66 (10)
C4—C3—C2	118.92 (13)	O1—C14—H14A	110.0
C4—C3—H3	120.5	C15—C14—H14A	110.0
C2—C3—H3	120.5	O1—C14—H14B	110.0
C6—C5—C4	120.49 (13)	C15—C14—H14B	110.0
C6—C5—H5	119.8	H14A—C14—H14B	108.3
C4—C5—H5	119.8	O3—C18—N2	123.61 (13)
C10—C11—C12	118.92 (13)	O3—C18—C19	122.07 (12)
C10—C11—H11	120.5	N2—C18—C19	114.31 (12)
C12—C11—H11	120.5	C1—C2—C3	120.07 (13)
C9—C8—C13	118.00 (12)	C1—C2—H2	120.0
C9—C8—C7	121.76 (12)	C3—C2—H2	120.0
C13—C8—C7	120.24 (12)	C3—C4—C5	121.37 (12)
O4—C1—C2	123.97 (12)	C3—C4—Cl1	119.56 (11)
O4—C1—C6	115.20 (12)	C5—C4—Cl1	119.08 (11)
C2—C1—C6	120.83 (12)	N2—C17—C16	111.17 (11)
C5—C6—C1	118.23 (12)	N2—C17—H17A	109.4
C5—C6—C7	121.01 (12)	C16—C17—H17A	109.4
C1—C6—C7	120.75 (12)	N2—C17—H17B	109.4
C8—C7—C6	113.54 (11)	C16—C17—H17B	109.4
C8—C7—H7A	108.9	H17A—C17—H17B	108.0
C6—C7—H7A	108.9	C11—C10—C9	121.58 (13)
C8—C7—H7B	108.9	C11—C10—Cl2	118.62 (11)
C6—C7—H7B	108.9	C9—C10—Cl2	119.77 (11)
H7A—C7—H7B	107.7	N1—C16—C17	110.63 (11)
O1—C13—C12	123.54 (12)	N1—C16—H16A	109.5
O1—C13—C8	115.16 (11)	C17—C16—H16A	109.5
C12—C13—C8	121.29 (12)	N1—C16—H16B	109.5
C8—C9—C10	120.29 (12)	C17—C16—H16B	109.5
C8—C9—H9	119.9	H16A—C16—H16B	108.1

### Hydrogen-bond geometry (Å, º)

Cg1 is the centroid of the C1–C6 ring.

**Table:**

*D*—H···*A*	*D*—H	H···*A*	*D*···*A*	*D*—H···*A*
N2—H1*N*2···O2^i^	0.89	2.05	2.8945 (15)	158
C14—H14*A*···O3^i^	0.97	2.54	3.5026 (17)	172
C14—H14*B*···O3^ii^	0.97	2.34	3.2934 (17)	167
C16—H16*B*···O1^iii^	0.97	2.59	3.2843 (17)	129
C19—H19*A*···Cl2^iv^	0.97	2.81	3.6201 (14)	142
C9—H9···*Cg*1^v^	0.93	2.88	3.8006 (14)	174
N1—H1*N*1···O1	0.90	2.16	2.5935 (15)	109

Symmetry codes: (i) −*x*+1, *y*−1/2, −*z*+1/2; (ii) *x*, −*y*+3/2, *z*−1/2; (iii) −*x*+1, *y*+1/2, −*z*+1/2; (iv) −*x*+2, *y*+1/2, −*z*+1/2; (v) −*x*+2, *y*−1/2, −*z*+1/2.
